# Review on Micro-Alloying and Preparation Method of 7xxx Series Aluminum Alloys: Progresses and Prospects

**DOI:** 10.3390/ma15031216

**Published:** 2022-02-06

**Authors:** Yuxin Dai, Liangming Yan, Jianpeng Hao

**Affiliations:** College of Materials Science and Engineering, Inner Mongolia University of Technology, Hohhot 010051, China; 20211000049@imut.edu.cn (Y.D.); jianpeng19980906@163.com (J.H.)

**Keywords:** 7xxx series aluminum alloys, micro-alloying, aging precipitation sequence, strengthening-toughening mechanism, preparation method

## Abstract

Notably, 7xxx series aluminum alloy has become the most popular nonferrous alloy, extensively used in industry, construction and transportation trades, due to its high comprehensive properties such as high static strength, high strength, heat resistance, high toughness, damage resistance, low density, low quenching sensitivity and rich resource. The biggest challenge for aluminum alloy today is to greatly improve the corrosion resistance of the alloy, while maintaining its strength. The preparation method of 7xxx series aluminum alloy requires controlling time lapses in the process of heating, holding and cooling, and there are many species precipitates in the crystal, but the precipitated strengthening phase is a single type of equilibrium η′ phase. Therefore, more attention should be paid to how to increase the volume fraction of η′ precipitates and modify the comprehensive performance of the material and focus more on the microstructure of the precipitates. This article reviews the progress of 7xxx series aluminum alloy materials in micro-alloying, aging precipitation sequence, the strengthening-toughening mechanism and the preparation method. The effect of adding trace elements to the microstructure and properties of 7xxx series aluminum alloy and the problems existing in aging precipitation characteristics and the reinforcement mechanism are discussed. The future development direction of 7xxx series aluminum alloy is predicted by developing a method for the process-microstructure-property correlation of materials to explore the characteristic microstructure, micro-alloying, controlling alloy microstructure and optimizing heat-treatment technology.

## 1. Introduction

The first generation of 7xxx super-hard aluminum alloy was investigated in 1930, and developers are now looking at fifth generation aluminum alloy materials. Notably, 7xxx series aluminum alloy can be strengthened by heat-treatment and can achieve 490–820 MPa [1,2,3,4,5]. According to the addition of elements, alloys are mainly classified into two categories: Al-Zn-Mg alloy has exhibited better weldability and general corrosion resistance, and high strength can be obtained when heat treatment is appropriate; the other is developed on the basis of Al-Zn-Mg alloy by adding Cu, which has high specific strength, low density, yield strength close to tensile strength, and exhibits better corrosion resistance and high toughness. It is the highest strength series of aluminum alloy and is easy to machine, and is applied widely in large aircraft manufacturing and aerospace, and internationally recognized as the main aviation material [6,7,8]. In this paper, the new principle of process-microstructure-performance correlation is used to continuously improve the microstructure-performance characterization method of aluminum alloy, based on the composition-process-microstructure-performance relationship of 7xxx series aluminum alloy, which shows good prospects for the future development direction of aluminum alloy.

## 2. Micro-Alloying

After adding Zr, Mn, V, Cr and other micro-elements to 7xxx series aluminum alloy, the hardness of the alloy decreases and the hardenability decreases, in turn, during slow quenching. In this section, the effects of four kinds of elements on the properties of 7xxx series aluminum alloy are discussed, respectively. Table 1 summarizes the related properties of Al-Zn-Mg-Cu alloy.

### 2.1. Main Alloy Elements

In Al-Zn-Mg alloy, Zn and Mg are the main alloy elements, comprising not more than 7.5%. With the increase in Zn and Mg content, the ultimate tensile strength and heat treatment effect improved. When the content of Mg + Cu ≤ 7%, the alloy exhibits better stress-corrosion resistance, and the welding crack tendency decreases with the increase in Mg. The main function of the Cu element in Al-Zn-Mg-Cu alloy is to improve the corrosion resistance of the material, and the Cu element also has a certain enhancement effect [19]. Zn and Mg will form η (MgZn_2_) phase and T (Al_2_Mg_2_Zn_3_) phase after co-existence and aging. Mg/Zn mass ratio in the range of 5:2–7:1 can refine the precipitates and improve the strength of the alloy; when the content of Zn + Mg ≥ 10%, the electrical conductivity, fracture toughness, stress-corrosion resistance and exfoliation corrosion resistance of the alloy begin to decrease [20].

When the content of Zn/Mg > 2.2 and Cu > Mg, the strengthening phase S (CuMgAl_2_) can enhance the strength of the alloy. The increase in Cu content will promote the recrystallization of the alloy, increase the density of the precipitated phase, and reduce the potential difference between the grain boundary and the grain [21]. The effect on PFZ (precipitation free zone) width is small, and the stress-corrosion resistance of the alloy is improved. If the element ratio is not within this range, S phase is the brittle phase that destroys the properties of the alloy. The size difference between the Cu atom and the Al atom is quite distinct; consequently, the crystal lattice deformation of Cu dissolved in Al-based solid solution in the form of replacement solid solution will reduce the hardenability of aluminum alloy and promote the precipitation of second phase in quenching [22]. In the process, the aging strengthening effect is reduced, and the quenching sensitivity of the alloy is reduced. The artificial aging process of the alloy in the range 100–200 °C is accelerated, expanding the stable temperature range of the GP (pre-dissolved atomic segregation area) region and improve the ultimate tensile strength, plasticity and fatigue strength. However, the addition of Cu tends to produce intercrystalline corrosion and pitting corrosion. The commonly used chemical composition of 7xxx series aluminum alloys is shown in Table 2 [20,21,23,24,25,26]. In addition to the main alloy elements such as Zn, Mg and Cu, there are also minor Mn and Cr elements that not only form new strengthening phases with the main alloy elements to increase the alloy strength, but also simultaneously improve the thermal strength and corrosion resistance.

### 2.2. Microalloying Elements

Adding microcontent of Zr, Mn, Cr, Ag elements in 7xxx series aluminum alloy can not only improve the recrystallization temperature of the alloy, but also refine crystal grain, thus giving the alloy high mechanical properties and anti-corrosion capacity.

#### 2.2.1. Zr and Mn Transition Elements

Fang et al. [24] found that the addition of Zr (0.1%~0.15%) in 7xxx series aluminum alloy can form Al_3_Zr dispersion phase with significant strengthening effect on the alloy. The pinning effect of Al_3_Zr on the grain boundary can inhibit the recrystallization and grain growth behavior of the alloy, and improve the yield strength, tensile strength and elongation of the alloy. Due to the extremely low solubility of Zr in aluminum, Al_3_Zr particles are easily precipitated with the addition of microcrystalline Zr element. It is concluded that Al_3_Zr is a small particle dispersed in the grain and on the crystal boundaries, which is beneficial to refine the alloy grain, improve the strength, toughness, aging effect and corrosion resistance of the alloy. 

There are only three ways for Zr to exist in aluminum alloy [25]: (1) Solid solution in the matrix; (2) In the process of solid solution or homogenization, coherent or incoherent Al_3_Zr (D0_23_) dispersion particles are released from the supersaturated solid solution, which acts as dispersion strengtheners; (3) During the melting process, Zr element segregates from the matrix, resulting in the formation of Al_3_Zr primary crystals with coarse size, which is not conducive to the alloy’s performance. 

Md Shahnewaz et al. [27] found that the addition of Mn in 7xxx series aluminum alloy forms Al_6_Mn particles improves the stress-corrosion resistance of the alloy. Al_6_Mn does not affect the width of PFZ and can increase the maximum tensile strength, fracture toughness, and simultaneously improve the low cyclic fatigue performance. Valeev SH I et al. [28] pointed out that excessive Mn content would lead to the emergence of Al_20_Cu_2_Mn_3_ and other second-phase compounds in aluminum alloys, which reduced the number of strengthening elements and strengthening phases in aluminum alloys, and the quenching sensitivity increased with the excess level of Mn content.

#### 2.2.2. Cr and Ag Microalloying Elements 

Notably, 7xxx series aluminum alloy has strong quenching sensitivity, and it is difficult to prepare rather thick plates with consistent strength [8,29]. If Zr is substituted with Cr, the quenching sensitivity can be greatly reduced and the effect of Cr on inhibiting recrystallization of 7xxx series aluminum alloy can be retained. Many investigations have shown that [24,30,31,32]: Cr element can form fine second phases of intermetallic compounds such as E (Al_18_Cr_2_Mg_3_) phase, (CrMn)Al_12_ and (CrFe)Al_7_ in aluminum alloy, which can effectively prevent the recrystallization nucleation and growth of aluminum alloy during processing. Micro amounts of Cr will improve the stress-corrosion resistance of aluminum alloy, and it will combine better with Cu. Generally, the content of Cr in aluminum alloy is 0.1–0.2%. However, in recent years, due to the better comprehensive performance of dispersion particles generated by Zr and other elements or aluminum matrix, Zr is used to replace Cr.

**Figure 1:**
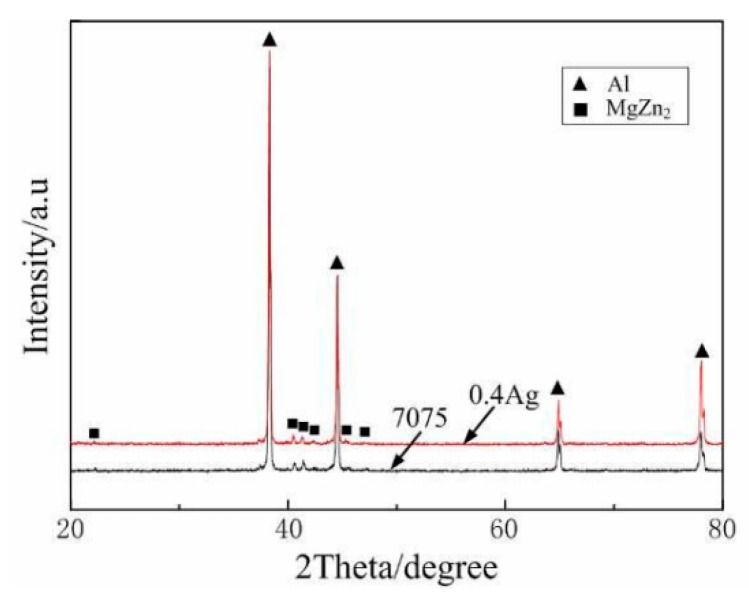
XRD pattern of as-cast 7075 (0 Ag) alloy and 0.4 Ag alloy. Adapted with permission from ref. [34]. 2015 Xu.

Adding minor Ag (0.16%) to Al-Zn-Mg-Cu-Zr alloy disclosed that Ag could promote the formation of GP zone and transition phase [33], improve the stability of transition phase, delay the over-aging of the alloy, and thus improve the thermal stability of aluminum alloy [34]. Figure 1 XRD shows that the addition of Ag element in 7xxx series aluminum alloy does not change the main phase composition.

**Figure 2:**
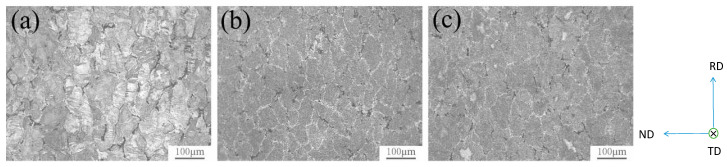
Microstructure of rolled alloys with different compositions: (**a**) 0 Ag (7075), (**b**) 0.2 Ag and (**c**) 0.4 Ag. Adapted with permission from ref. [34]. 2015 Xu.

Figure 2 shows the rolled structure of the alloy with different content of the Ag element. After homogenization treatment and rolling deformation of 7075 alloy, it can be seen that the grains become longer along the rolling direction. After homogenization treatment, there are still superior second phases around the grains. The second phase of the alloy containing Ag is less than that of the alloy without Ag, and the aggregation phenomenon is weakened.

### 2.3. Rare Earth Elements

There are 17 rare earth elements, which are the third subfamily of the periodic table. The addition of rare earth elements can purify, modify, micro alloy, and strengthen and improve the electrical conductivity of aluminum alloys. Depending on the different performance needs of aluminum alloys, the mass fraction of rare earth elements in the range of 0.1–0.4% is the best proportion [35]. Table 3 [20,24,36,37,38] explains the characteristics of the main rare earth elements in aluminum.

It can be discerned from Table 3 that the difference between the melting point of pure aluminum and the eutectic temperatures of rare earth elements Er and Sc is relatively low, and the eutectic point composition of Sc is the lowest, w (Sc)% ≈ 0.3%, w (Er) ≈ 1.0%. Therefore, it can be perceived that the properties of 7xxx series aluminum alloy with the addition of Sc will be remarkably improved.

#### 2.3.1. Er

Liu et al. [39] identified the effect of rare earth element Er on the failure behavior of 7xxx series aluminum alloy. The results show that the addition of Er can enhance the strength of 7xxx series alloy. When Zr and rare earth elements coexist, the performance of the alloy gets better. When the mass fraction of Zr is 0.14%, Er has a strong ability to improve strength (4.2%). When the mass fraction of Zr is 0.27%, Er has a weak ability to improve strength (3.1%); a trace addition of Er can improve the plasticity of the material; the fracture mechanism of the alloy without Er is brittle fracture, and secondary cracks appear. The toughness of the alloy with a trace addition of Er is enhanced, and dimples appear, which changes from brittle fracture to ductile fracture mode [40]. 

#### 2.3.2. Sc

Due to the decrease in the recrystallization fraction and the discontinuous distribution of GBPs, 0.06 wt% Sc added to medium strength Al-Zn-Mg alloy has excellent SCC resistance under aging conditions [41]. Excessive addition of 0.11 wt% Sc can increase the electrochemical activity and hydrogen embrittlement rate of GBPs and PFZ, thereby reducing the SCC resistance of the alloy.

**Figure 3:**
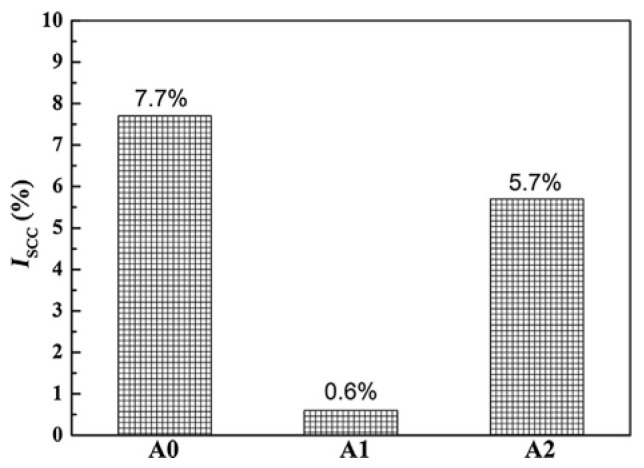
SCC susceptibility index (ISCC) of three peak-aged alloys. For Al Zn Mg alloys with 0 wt% Sc (A0), 0.06% Sc (A1) and 0.11% SC (A2)After homogenization treatment (470 °C for 24h), the ingot is preheated at 450 °C for 1H. Adapted with permission from ref. [41]. 2018 Li et al.

**Figure 4:**
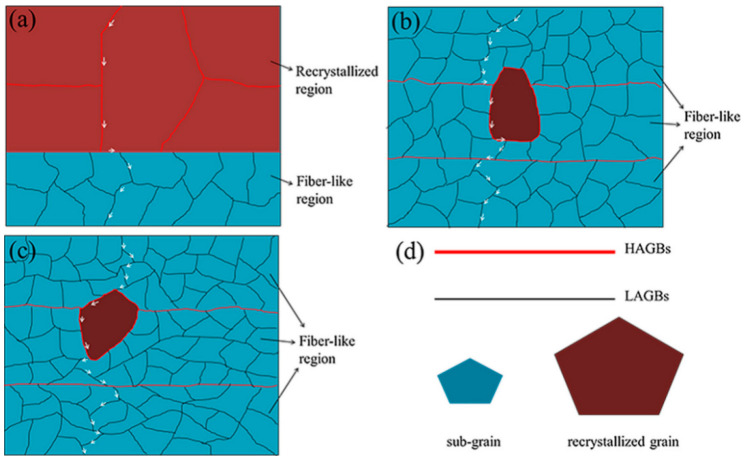
Schematization of SCC crack propagation paths for three peak-aged alloys: (**a**) A0; (**b**) A1; (**c**) A2; (**d**) the type of GB and grain. Adapted with permission from ref. [41]. 2018 Li et al.

Figure 3 exhibits the peak SCC sensitivity index (ISCC) of the three alloys. Figure 4 shows the SCC crack growth path diagram of the three alloys. Due to the decrease in the recrystallization fraction and the discontinuous distribution of GBPs, 0.06 wt% Sc added to medium strength Al-Zn-Mg alloy has superordinary SCC resistance and excellent mechanical properties, even under aging conditions. Excessive addition of 0.11 wt% will increase the electrochemical activity and hydrogen embrittlement rate of GBPs and PFZ, and reduce the SCC resistance of the alloy.

#### 2.3.3. Sc and Zr

The addition of trace amounts of Sc and Zr in Al Zn-Mg-Cu alloy is more beneficial for improving the extremely fine Al_3_ (Sc, Zr) particles, and the alloy obtains higher strength and ductility, because the dispersed Al_3_ (Sc, Zr) particles with L1_2_ structure are formed during homogenization. Al_3_ (Sc, Zr) particles with L1_2_ structure precipitates at any time into coherent Al_3_ (Sc,Zr) particles with D0_23_ structure. L1_2_ structure precipitated can effectively hinder recrystallization, inhibit grain growth, and improve the mechanical properties of the alloy [42]. Sc is a transitional element in rare earth elements and has been used as a kind effective grain refiner to replace Ti/TiB. Primary Al_3_Sc particles can be used for excellent heterogeneous nucleation and can effectively refine the grain size of aluminum alloy during solidification. In the Al−Sc binary system, the recrystallization can be effectively retained by adding between 0.15 and 0.20% Sc. Xiao et al. [43] found that compared with the addition of 0.10% Zr, the alloy with 0.07% Sc and 0.07% Zr can resist recrystallization more effectively and improve the mechanical properties of 7xxx series aluminum alloy.

#### 2.3.4. Y

In recent years, it has been found that a small amount of Y can refine the secondary dendrites of 7xxx alloy, reduce the size of eutectic compounds and improve the impact toughness of the alloy. According to the composition of the main alloy elements, Al_3_Y, Al_6_Cu_6_Y or Y_12_Al_3_Zn rare earth compounds will be formed, which have an obvious effect on grain refinement. Li et al. [44] found that after adding 0.25 Er and 0.15 Y to 7xxx series aluminum alloy, the dissolution temperature of eutectic compound increased, the nucleation rate increased, and the fine grain strengthening was achieved. Table 3 points out that the atomic radius difference between the Y atom and the Al atom is 26%. The deformation mechanism of Y in 7xxx series aluminum alloy is that when the Y atom enters the aluminum alloy, the lattice distortion and the free energy of the alloy increase, and the rare earth phase compounds containing Y can only be distributed at the grain boundary. Furthermore, when Y enters the alloy, the second phase at the grain boundary increases, which inhibits the growth of grains. The generated Al_3_Y, Al_6_Cu_6_Y or Y_12_Al_3_Zn rare earth compounds as the core of heterogeneous nucleation can strengthen the aluminum alloy. If the rare earth element Y is excessive, the second phase will be enriched, and the comprehensive properties of the alloy will be greatly reduced. Due to the low price of rare earth element Y, it is an important additive element with potential for future development.

#### 2.3.5. Gd

The rare earth element Gd also has a certain influence on the high temperature properties of the alloy. The addition of rare earth element Gd to the 7xxx series aluminum alloy forms a uniformly distributed L1_2_-type Al_3_ (Gd, Zr) dispersed phase, rather than a core-shell-shaped Al_3_ (Sc, Zr) dispersed phase produced by adding Sc element to the 7xxx series aluminum alloy. Chen et al. [45] found that the addition of 0.11% Gd in 7xxx series aluminum alloy has a significant effect on hindering dislocation and grain boundary movement, stabilizing a large number of deformation and recovery structures of fine sub-grain boundaries. The stress-corrosion crack propagation rate of the alloy was effectively delayed. The addition of 0.11% Gd element to 7056 aluminum alloy also increased the KI (critical stress intensity factor) of 7056 from 5.45 MPa·m^1/2^ to 10.59 MPa·m^1/2^.

**Figure 5:**
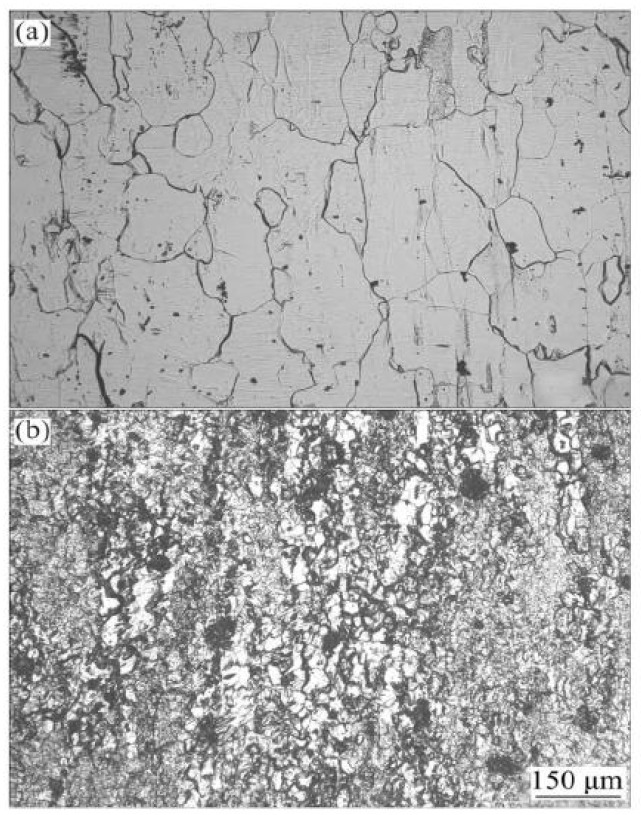
Optical microstructures of the two studied alloys with solution treatment: (**a**) 0% Gd and (**b**) 0.25% Gd. Adapted with permission from ref. [46]. 2012 Mei et al.

Mei et al. [46] identified, as shown in the Figure 5, that after high temperature solution treatment, the alloy without Gd added fully recrystallized, forming equiaxed recrystallized grains, and the alloy with Gd added still maintains fine fibrous unrecrystallization microstructure, indicating that the formed dispersion phase can effectively hinder the transformation of deformation-recovery microstructure to sub-grain microstructure, thereby inhibiting the recrystallization of the matrix. This shows that the rare earth element Gd is mainly distributed among dendrites in 7075 aluminum alloy with Al_3_Gd compound. Adding Gd within the effective range can improve the tensile strength and elongation of aluminum alloy.

### 2.4. Non-Metallic Inclusions Element

Fe and Si elements are harmful impurities in 7xxx series aluminum alloys. The main phases of impurity elements Fe and Si in 7xxx series aluminum alloys are brittle phases such as Al_7_Cu_2_Fe, MgSi_2_, AlFeMnSi and eutectic compounds [20,24]. Due to the impurity particles containing Fe and Si being distributed inside the grains or on the grain boundaries, and they are difficult to dissolve at high temperature, it easily produces the banded structure that is arranged intermittently along the deformation direction during hot deformation. In the process of plastic deformation, due to the uncoordinated deformation of the brittle phase matrix, microcracks are easily generated on some grain-matrix boundaries and become the source of macroscopic cracks, which have an unfavorable influence on the plasticity and fracture toughness of the alloy. At present, the mass fraction of Fe and Si impurities in 7xxx series aluminum alloys should be limited to below 0.15% [20,24].

## 3. Aging Precipitation Sequence and Strengthening-Toughening Mechanism of 7xxx Series Aluminum Alloy

The main phases that can be brought about between the elements of Al-Zn-Mg-Cu quaternary alloy mainly include α phase (Al matrix), θ phase (CuAl_2_), S phase (Al_2_CuMg), η phase (MgZn_2_), β phase (Al_8_Mg_5_) and T phase (Al_2_Mg_2_Zn_3_). The precipitated phases affect the main properties of the material [47].

### 3.1. Precipitated Sequence

**Figure 6:**
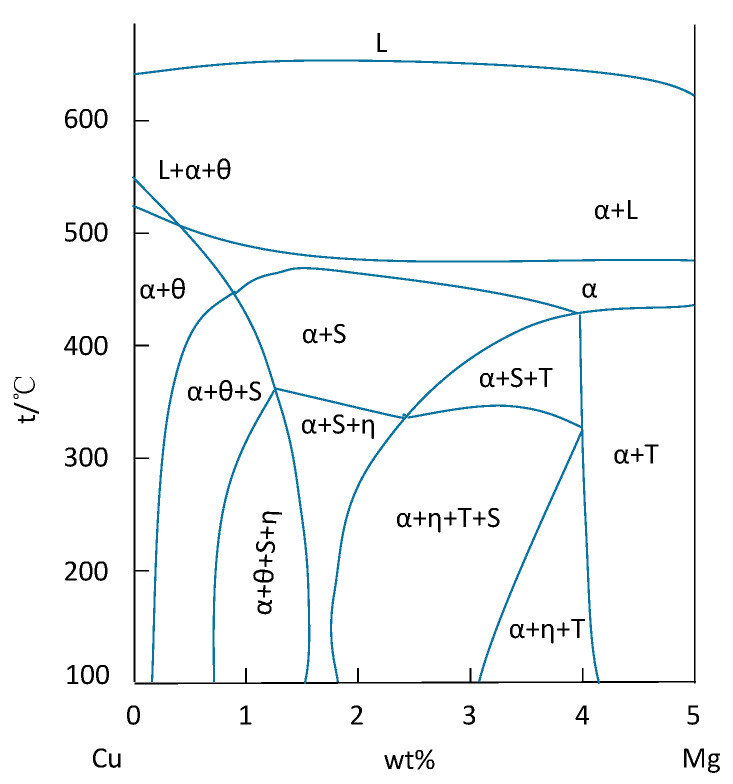
Variable temperature section of aluminum rich angle of equilibrium phase of 7xxx series aluminum alloy. Adapted with permission from ref. [48]. 2018 Zhang and Deng.

Figure 6 [48] shows the temperature dependence of Al-rich angle of equilibrium phase in 7xxx series aluminum alloys. According to the phase diagram, the increase in Cu content promotes the appearance of θ phase. When the Cu content is lower, the phase composition is mainly affected by the Zn/Mg ratio. When the Zn/Mg ratio is extremely small, all the amounts are composed of α + S + T phase or medium α + T phase without η phase; when the Zn/Mg ratio increased, η phase began to appear and gradually increased, accompanied by S phase and T phase.

**Figure 7:**
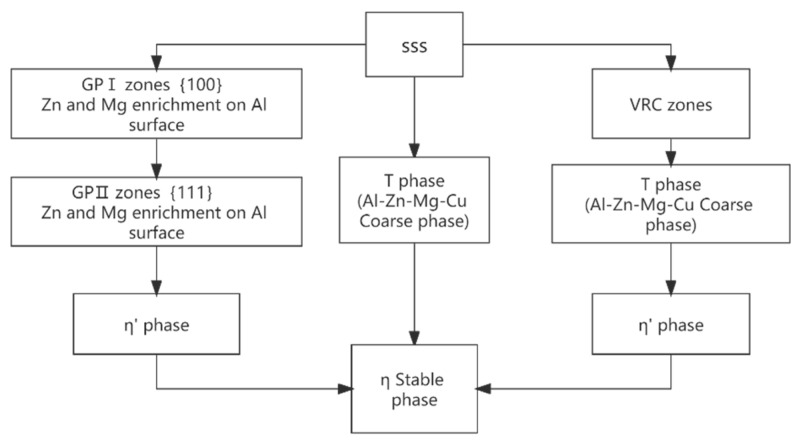
Precipitation sequence of precipitates in 7xxx series aluminum alloy. Adapted with permission from ref. [48]. 2018 Zhang and Deng.

The precipitation sequence of precipitates in 7xxx series aluminum alloy is classified into three types: the first is GP zone (supersaturated solid solution SSS → GP I zone → GP II zone → η′ phase → η phase); the second is when the Mg content is high, supersaturated solid solution SSS appears in defects and grain boundaries, the precipitation order is: (supersaturated solid solution SSS → T phase and macroscopic Al-Zn-Mg-Cu phase → η phase); finally, when the supersaturated solid solution SSS appears in the vacancy-rich region, the precipitation order is (SSS → VRC (vacancy-related cluster) vacancy enrichment → T phase and coarse Al-Zn-Mg-Cu phase → η phase). The three kinds of different precipitation behavior eventually formed a stable η phase; the precipitation order is shown in Figure 7 [48]. The main strengthening phases of Al-Zn-Mg-Cu alloy include GP zone and η′ phase. The GP zone is a fully coherent Mg and Zn enrichment zone with the aluminum matrix, which is spherical (GP I zone) or strip (GP II zone), while the η′ phase is a hexagonal plate metastable phase, semi-coherent with the aluminum matrix, which is the uppermost aging strengthening phase. The η phase is a disc-shaped equilibrium phase that is not coherent with the matrix [49]. If there is T phase under certain composition conditions, η phase will be replaced by T phase. Due to the existence of Cu atoms in the alloy, the S phase will appear at the grain boundaries at a certain Zn/Mg ratio. It will act as a cathode to continuously dissolve the surrounding matrix to form pitting corrosion, which will also lead to poor fracture properties of aluminum alloys. Table 4 summarizes the main precipitated phases and their structure characteristics in 7xxx series aluminum alloys [1,6,48,49].

**Figure 8:**
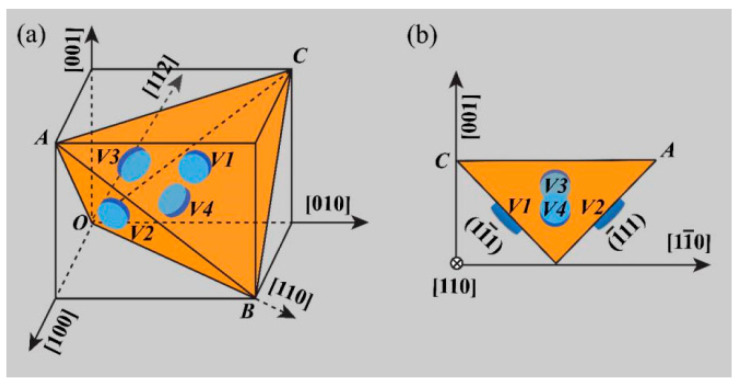
Schematic illustrations showing four types of η variants V1-4 on {111}_Al_ (**a**) The three-dimensional view, and (**b**) the projection from {110}_Al_ direction. Adapted with permission from ref. [55]. 2021 Zang et al.

According to the crystallography theory [11,55], there are four equivalent variants of η phase on the Al plane. As shown in Figure 8a, four disk η phases with the same thickness and diameter keep their (0001)η habit planes parallel to four equivalent {111}_Al_ planes, which are defined as V1–V4 variants. Figure 8b shows that V1 and V2 variants are marginal, while V3 and V4 variants are elliptical, as observed in the direction {110}_Al_. It can be seen from Figure 8 that when the temperature is lower than 100 °C, the η precipitates in the alloy are mainly V3 and V4. When the temperature range is 125–175 °C, V1 and V2 mainly contain η precipitates [55].

**Figure 9:**
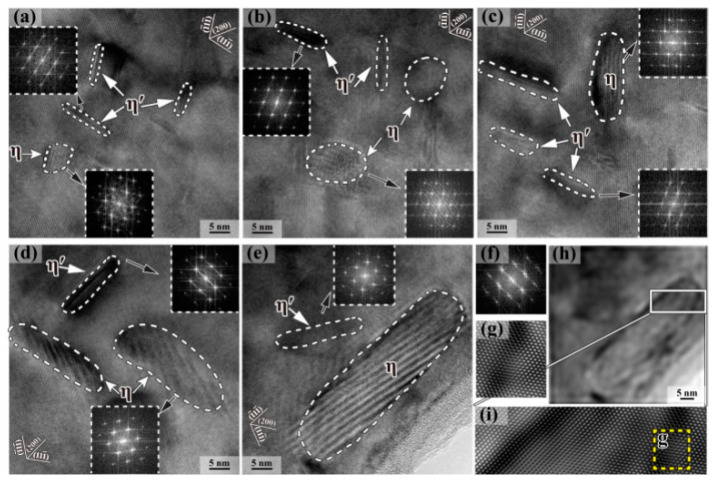
HRTEM images of the precipitates viewed along <110> Al orientation in 7085 alloy after various heat treatments: (**a**) sample I (25 °C); (**b**) sample II (100 °C); (**c**) sample III (125 °C); (**d**) sample IV (150 °C); (**e**) sample V (175 °C); (**f**) corresponding FFT pattern of η phase in (e); (**g**) is from the yellow dashed frame in (i); (**h**) is the corresponding inverse FFT image of (**e**), and; (**i**) is from the white solid frame in (h). Adapted with permission from ref. [55]. 2021 Zang et al.

Figure 9a shows that η′ phase is thin margin (elongated) on {111}_Al_ plane. The insertion of the upper left corner shows the fast fourier transform (FFT) pattern diagram of the η′ phase with weak scattering. When the temperature reaches 175 °C, Figure 9e, η′ thickness and radius increase, respectively. A larger radius and thickness of η precipitates can also be observed. The clearer observation results in Figure 9g show the obvious aboriginal lattice distortion caused by the incoherence between η phase and α-Al matrix.

**Figure 10:**
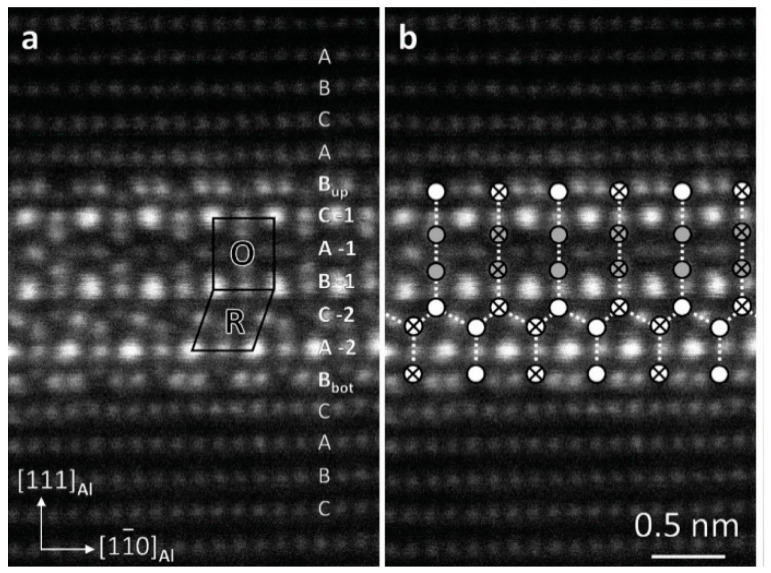
Lattice and crystal structure of 7xxx series aluminum alloy under haddf stem (**a**) STEM image of the η precipitate showing the continuity of stacking of the fcc aluminium lattice. (**b**) Overlay of the structure’s skeleton made up from the major ligands. Adapted with permission from ref. [58]. 2020 Bendo et al.

Khalfallah et al. [56] found by DSC that the formation of GP region is controlled by the migration of Zn and Mg atoms, while η′ metastable and η precipitation of stable phase is affected by the migration and diffusion of solute atoms. Berg et al. [57] found that GP I region was formed in a wide temperature range from room temperature to 140–150 °C, and was independent of quenching temperature. These regions are consistent with the aluminum matrix. Based on the AlCu_(I)_ type subunit, the interior of the matrix lattice is arranged as Zn and Al or Mg, and the periodic inverse boundary is formed after quenching above 450 °C and aging above 70 °C. According to the diffraction theory, the GPII region is identified as a Zn-rich layer on the {111} plane, the inside of which is arranged in a slender crystal orientation. The habit plane of GPII region is the {111} plane, indicating that not all GP regions can be transformed into η′ phase. The η′ phase is seven-layer {111}_Al_ thick, with O (orthorhombic) and R (rhombohedral) subunit units inside. Each ligand is formed by the push-ring contact between two atoms and six atoms. It can be seen that the GP zone with smaller size has higher surface energy, which is more unstable than the GP zone with larger size. With the aging process, the GP zone with smaller size is transformed into η′ phase. Figure 10 [58] shows the lattice and crystal structure of 7xxx series aluminum alloy under haddf-stem.

### 3.2. Strengthening and Toughening Mechanism

#### 3.2.1. Strengthening

**Figure 11:**
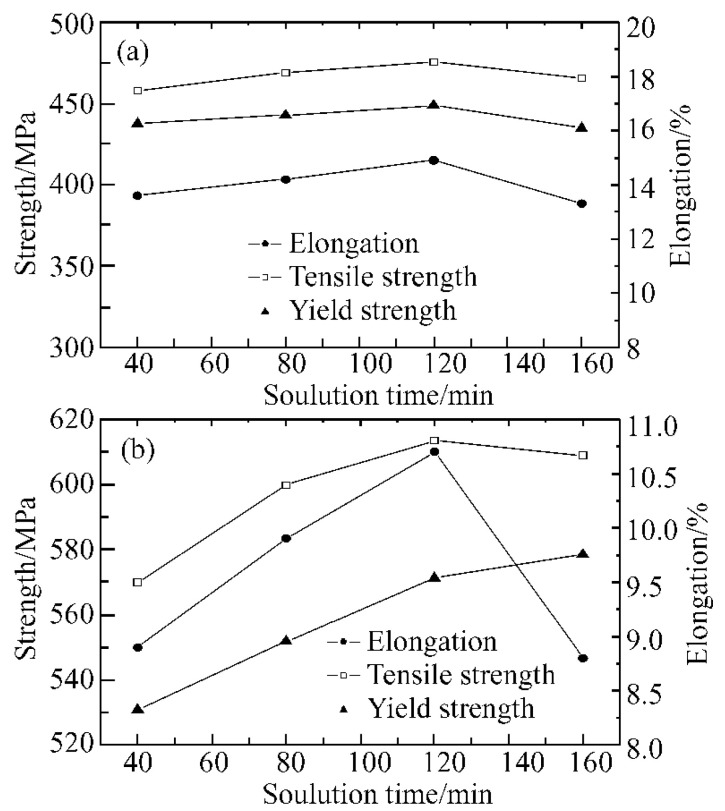
Change of tensile properties of studied alloy with solution time at 470 °C (**a**) solution (**b**) solution+T6. Adapted with permission from ref. [64]. 2007 Dai et al.

The strengthening and toughening of 7xxx series aluminum alloy deformation mainly includes solid solution strengthening, second phase strengthening, grain boundary strengthening and processing strengthening. The existing problem is still that the comprehensive performance of strength and toughness and stress-corrosion resistance are low. According to the aging precipitation [55,59,60,61,62], the phase, GBPs and PFZ of the precipitated alloy after aging determine the properties of the aluminum alloy. Reducing the slip of the eutectic surface and narrowing the PFZ of the grain boundary, optimizing the composition design of the alloy and improving the heat treatment method are the specific means to improve the comprehensive properties of the alloy [46]. Improving the properties of the aluminum alloy cannot only start from the composition design, but also must understand the performance theory and characterization method of the microstructure in depth [63]. Dai et al. [64] researched the mechanical properties of 7xxx series aluminum alloy at 470 °C under different solution times. With the extension of solution holding time, the tensile strength and elongation of the alloy increased first to the peak and then decreased gradually. Under 470 °C solution state, the tensile strength and yield strength of the alloy were the highest when the aging time was 120 min, which were 475.3 MPa and 448.6 MPa, respectively. After solution treatment and aging at 120 °C for 24h, the tensile strength reached a peak of 613.5 MPa at 120 min, and the yield strength reached a peak of 578.5 MPa at 160 min. Figure 11 shows the changes of mechanical properties of the alloy at the peak point of tensile strength and yield strength at 470 °C for different solution times.

When the second phase is uniformly distributed in the matrix with fine dispersed particles, the precipitation strengthening or aging strengthening (dislocation cutting through the second phase mechanism) occurs when the second phase precipitates and produces a strengthening phase through the aging treatment of the supersaturated solid solution. If the second phase particles are strengthened by powder metallurgy, it is called dispersion strengthening [61,65]. The elements of the material composition affect the strength of the second phase particles, grain boundaries, grain size and orientation, grain boundaries and separation, and residual stress in the material. The formation of the second phase particles belongs to the diffusion-type phase transformation. When the dislocation meets the metastable precipitates such as η′ (MgZn_2_) phase, η_p_ (Al_2_Mg_2_Zn_8_) phase, S′ (Al_2_CuMg) phase, β′ (Al_8_Mg_5_) phase and T′ (Al_2_Mg_2_Zn_3_) in 7xxx series aluminum alloy, the dislocation will cut through the above metastable precipitates and deform with the matrix at the same time. The strength of the alloy is improved by increasing the interface energy between the second phase particles and the interface. When the dislocation meets the precipitated phase η with larger steady-state size, it is incoherent with the matrix. The dislocation will be subjected to the second-phase particles to make the dislocation line bend larger and form the second-phase particle dislocation ring. Other dislocations bypass the second-phase particles. The effect of the bypass mechanism on strengthening is enhanced with the increase in particles and the decrease in the size [62].

**Figure 12:**
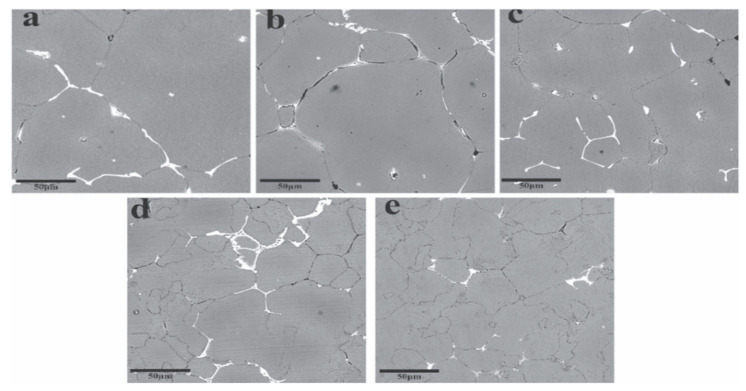
SEM images of 7055 as-cast alloys (**a**) Al-Y-0Sc, (**b**) Al-Y-0.2Sc, (**c**) Al-Y-0.25Sc, (**d**) Al-Y-0.3Sc, (**e**) Al-Y-0.35Sc. Adapted with permission from ref. [66]. 2021 Huang et al.

Kai Huang et al. [66] found that when Sc content was up to 0.25%, the grain dispersion strengthening refinement effect was effective, the particle size was more uniform, and the secondary dendrite spacing decreased. When the amount added was more than 0.30%, no grain refinement effect was observed, as shown in Figure 12.

**Figure 13:**
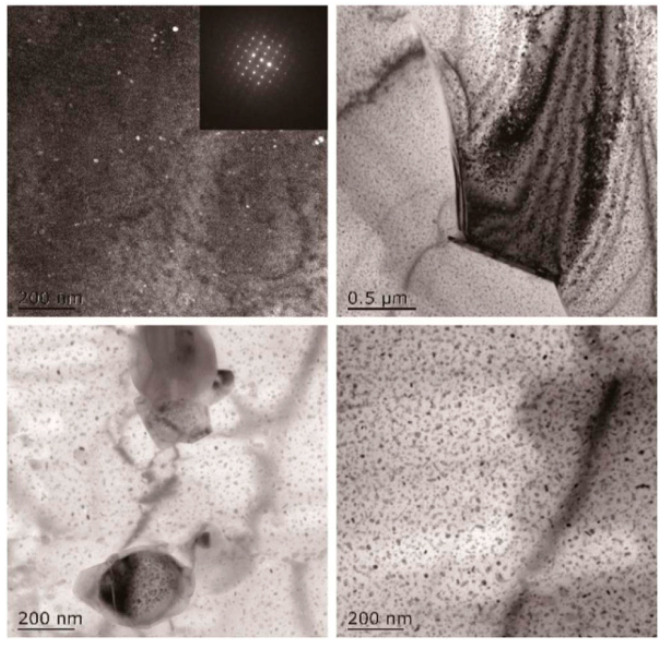
TEM topographic images of Al-0.25Y-0.25Sc alloy after aging. Adapted with permission from ref. [67]. 2018 Yan et al.

Figure 13 shows the high density Al (Sc_x_Y_y_) particles in Al-0.25Y-0.25Sc alloy grains, some of which are located in the dislocation position near GBs. The precipitates first precipitate and grow at GBs, and then transform into a stable phase by absorbing the solute atoms around the particles [67]. The second phase particles of Al_3_(Sc_x_Y_y_) can prevent the movement of substructure and dislocation, and as the alloy has the smallest particle size, its corrosion resistance is the best. Zhang et al. [68] found that the second phase particles could inhibit the precipitation and aggregation of θ-CuAl_2_ phase at GBs, which greatly reduced the corrosion sensitivity of the alloy. When the η′ (MgZn_2_) phase is transformed into η phase, the strength and plasticity of the alloy decrease instead. The η phase distributed at the grain boundary will cause the formation of PFZ. Dislocation and stress concentration can lead to the expansion of PFZ into cracks [69].

Both grain-boundary strengthening and processing-strengthening strengthen the material by changing the grain size. The prominent role of melting, and grain refinement, in improving the mechanical properties of aluminum products are two key issues [70]. The most obvious improvement in grain refinement is the increase in strength at room temperature, which can be explained theoretically by the Hall–Petch formula. In the polycrystalline, the yield strength is transferred from the grain with prior plastic deformation to the adjacent grain with slip. This transfer is reflected in the stress concentration generated by the dislocation accumulation group near the grain boundary of the slip grain. When the applied stress and other conditions reach a certain value, the number of dislocations is proportional to the distance between the obstacle causing the accumulation and the dislocation source at the grain boundary. Therefore, the larger the grain is, the larger is the distance, the larger the number of dislocations, and the greater is the stress concentration. The opportunity for plastic deformation is much larger than with small grains. This is why ductility increases with grain coarsening and decreases with grain size decreasing [62,70]. Compared with the second phase strengthening and deformation hardening, the grain boundary strengthening can improve the strength, toughness and ductility, and reduce the defects such as separation and porosity of castings [71].

#### 3.2.2. Strengthening and Toughening Method

The strengthening and toughening methods of 7xxx series aluminum alloys should not be limited to the simple design of the alloy composition in the “stir-frying style”. We should focus more on the microstructure of the precipitates and the microstructure improvement methods after heat treatment. More attention should be paid to how to eliminate S (Al_2_CuMg) phase, θ (CuAl_2_) phase and T (Al_2_Mg_2_Zn_3_) phase, and how to control the content of Mg/Zn to reduce the incoherent precipitation caused by η′ (MgZn_2_) phase. The addition of Zr and Sc atoms can form the dispersed Al_3_(Sc,Zr) particles that are coherent with the matrix, which can refine the grains and inhibit the recrystallization of the grain boundary. However, the transformation of Al_3_(Sc,Zr) particles from coherent to non-coherent should be avoided during the migration of the grain boundary. The non-coherent second phase particles will make the stress-corrosion resistance of the material worse and the quenching sensitivity higher.

**Figure 14:**
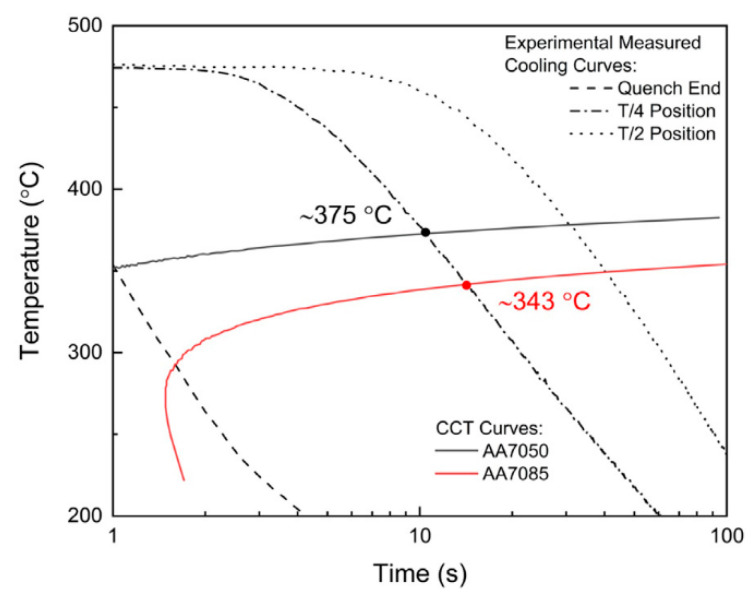
CCT curves for nucleation of η-phase Q-GBPs in AA7050 and AA7085. Adapted with permission from ref. [72]. 2021 Garner et al.

**Figure 15:**
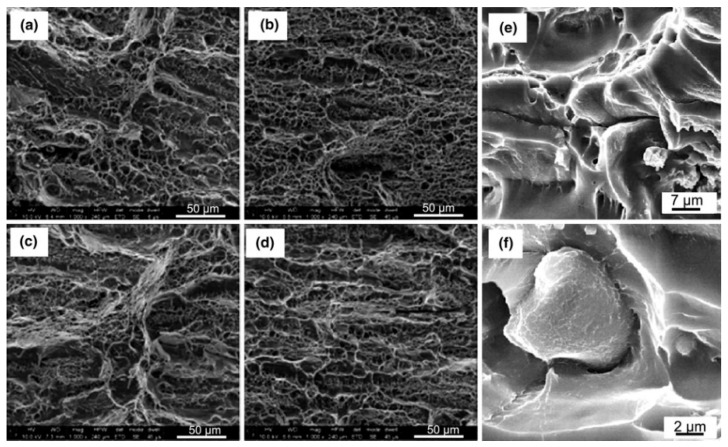
Fracture surface morphologies of AA7085 Al alloy under conditions of T6 (**a**), T74 (**b**), RRA (**c**), and HLA (**d**), temper conditions; high magnification images of fracture surface of AA7085 Al alloy show the cracking along the grain boundary triple points, dimples, and voids (**e**), cracking around coarse intermetallic particles (**f**). Adapted with permission from ref. [73]. 2010 Menzemer et al.

Furthermore, the method of strengthening and toughening aluminum alloy should be combined with theoretical simulation and experiment, focusing on the optimization of 7xxx series aluminum alloy deformation processing and heat treatment on the microstructure and stress changes. Alistair Garne et al. [72] found the quenching of 7050 and 7085 aluminum alloy η phase nucleation simulation, shown in Figure 14, and obtained the quenching process between the two alloys’ q-GBPs (quenching state) and a-GBPs (aging state) composition in the volume composition difference. Menzemer et al. [73] studied the fracture surface morphology of 7085 at various temperatures, as shown in Figure 15. The alloy quenched in cold water showed the main intergranular fracture characteristics. When quenching in oil medium, the fracture surface transforms into a combination of transverse and intergranular fracture. It can be seen that heat treatment methods such as hierarchical solid solution and aging can promote the dispersion and homogenization of η′ phase, and hinder the coarsening of PFZ region, thereby improving the strength and toughness of the alloy [74].

#### 3.2.3. First-Principle Calculation of Precipitation Strengthening Phase

**Figure 16:**
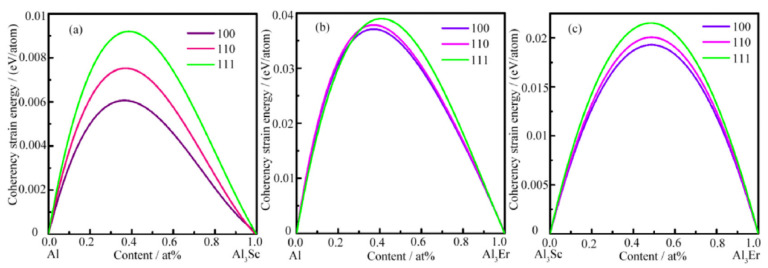
Coherency strain energies of (**a**) Al/Al_3_Sc, (**b**) Al/Al_3_Er, (**c**) Al_3_Sc/Al_3_Er on (100), (110), (111) interface planes. Adapted with permission from ref. [75]. 2021 Liu et al.

The first principle is based on the density functional theory (DFT), using the relativistic corrected projection augmented wave (PAW) method to describe the virtual element ratio; the crystal structure of the precipitated phase can be calculated, and the stability of the crystal structure can be judged. Liu et al. [75] calculated the interface energy of Al/Al_3_Sc, Al/Al_3_Er and Al_3_Sc/Al_3_Er in Al-Sc-Er alloy in three directions, based on the PBE function in VASP software. It was found that the interface structure of (100) surface was the best, and the interface energy of Al/Al_3_Er was the largest, as shown in Figure 16. The L_12_-Al_3_Sc_x_Er_1-x_ precipitates mainly formed a core-shell structure, with Al_3_Er as the core, and Al_3_Sc as the shell.

**Figure 17:**
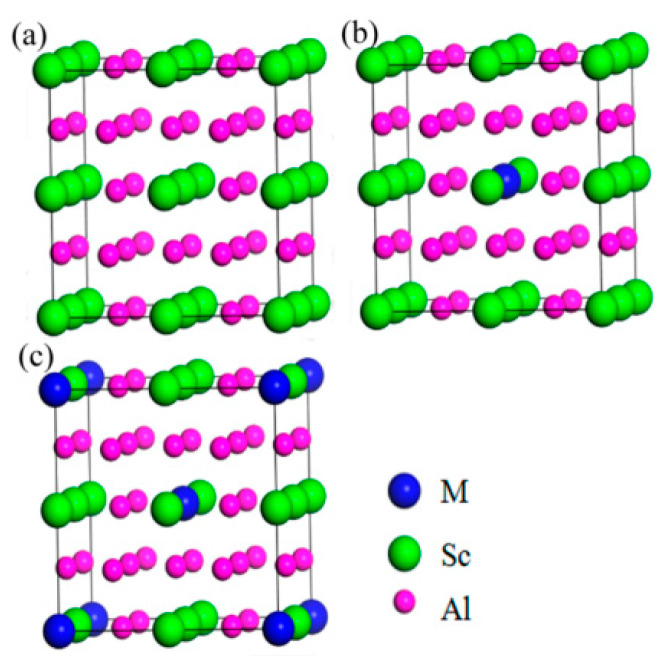
Structures of Al_3_Sc cell structure of alloy elements (M = Sc, Zr, Ti, Y, Li) doped with different doping concentrations: (**a**) 2 × 2 × 2 supercell; doped with the alloying element (M = Sc, Zr, Ti, Y, Li) at different doping concentrations, (**b**) 3.125%, and (**c**) 6.25%. Blue, green, and pink balls represent (M = Zr/Ti/Y/Li), Sc, and Al atoms, accordingly. Adapted with permission from ref. [77]. 2013 Sun et al.

Dong et al. [76] discussed the effect of doping elements (M) on the structural stability and mechanical properties of Al_3_Sc doped with Zr, Ti, Y and Li, as shown in Figure 16. The calculation shows that the structural stability, elastic properties and anisotropy of Al_24_Sc_6_Zr_2_ and Al_24_Sc_6_Ti_2_ are superior. Sun et al. [77] calculated that the point defects of L1_2_-Al_3_Sc were mainly Al vacancies and Sc antisite defects on the Al sublattice, as shown in Figure 17. Various mechanical properties of the material were calculated by the first principle method and the mechanical equation, which was convenient for the study of the total amount and proportion of the main alloy elements of the aluminum alloy, and was a new direction in the field of material design [61].

## 4. Preparation Method of 7xxx Series Aluminum Alloy

**Figure 18:**
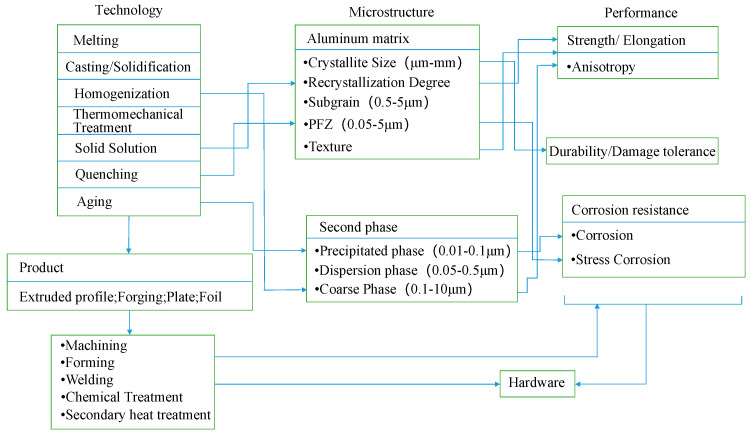
Technology microstructure performance relationship of 7xxx series aluminum alloy. Adapted with permission from ref. [79]. 2019 Deng and Zhang.

With regards to 7xxx series aluminum alloy, Fe and Si elements are considered as a natural impurities; solid solubility at room temperature is extremely low for insoluble intermetallic particles; insoluble intermetallic particles only through deformation and heat treatment alter the morphology, crystal type and composition [78]. All coarse intermetallic particles are principal formed in the process of direct cold casting. The non-uniformity of alloy is the difficulty and critical in the design and preparation of large-scale materials. For avoiding performance loss in the manufacturing process. The integrated molding of materials is also a development trend. The sophisticated formation mechanism of macroscopic, microscopic and multi-scale structures involved in the relationship of alloy composition-process-structure-performance is the main reason for determining the properties of materials [79,80]. Figure 18 shows the process-microstructure-performance relationship of 7xxx series aluminum alloy.

### 4.1. Casting Processes

The commonly used casting process for 7xxx series aluminum alloy is assigned to semi- continuous casting and squeeze casting. The casting structure mainly consists of primary α-Al, MgZn_2_, AlCuMg, Al_2_Cu and other second phases. With the decrease in pouring temperature, the primary α-Al dendrites in the slurry gradually decrease, and the near-isometric primary phase gradually increases. The grain size decreases, and the average roundness increases. In the solidification process of casting, the equilibrium or metastable phase formed by liquid-solid eutectic reaction occurs [80,81,82]. Bai [83] measured the chemical-mechanical properties of semi-solid 7050 alloy by in situ solidification method. It was found that the tensile strength and plasticity of the alloy initially decreased sharply with the decrease in solid fraction, which was due to the sharp decrease in the degree of intercrystalline polymerization of the semi-solid alloy with the increase in liquid phase. The tensile strength of the alloy decreased only slightly while the plasticity increased significantly. Thus, in the casting process it is most important to avoid macro segregation, cracks and other defects.

### 4.2. Multistage Homogenization Heat Treatment of Ingot

Firstly, 7xxx series aluminum alloy has a high degree of alloying, and the dendrite segregation of chemical composition in the ingot is especially obvious. The ingot of this alloy must be homogenized in the processing to eliminate the dendrite segregation of alloying elements and low melting point non-equilibrium eutectic phase, and reduce the heterogeneity of composition and structure. In practical engineering, the melting point of the non-equilibrium eutectic phase is usually measured by differential thermal analysis (DTA) or the differential scanning calorimetry (DSC) curve, and the melting temperature below the melting temperature of non-equilibrium eutectic phase is selected as the starting temperature of homogenization [84].

Secondly, another purpose of homogenization heat treatment is to regulate the behavior of high melting point precipitates of microalloying elements. The homogenization temperature of 7xxx alloy ingots is usually below 470 °C, because 480 °C is considered to be the over-burning temperature of 7xxx series aluminum alloy [79,85], at which time a large amount of residual components may still exist. In the case of large and thick aluminum alloy sheets or shaped ingots, it is difficult to synchronize the lifting and cooling rates, so it cannot be optimized only by single-stage homogenization. Wang et al. [86] found that the highest temperature of two-stage homogenization treatment of 7B04 aluminum alloy can reach 500 °C. The most appropriate homogenization heat treatment process is heating at 10 °C/h to 470 °C for 64 h, and then heating at 1 °C/h to 500 °C for 10 h. By comparing the non-equilibrium solidification components of 7B04 aluminum alloy ingot after ultra-high temperature homogenization at 500 °C, they were completely dissolved in the alloy matrix; the hot rolling plasticity is much better than that of the traditional hot rolling plasticity after homogenization at 470 °C.

**Figure 19:**
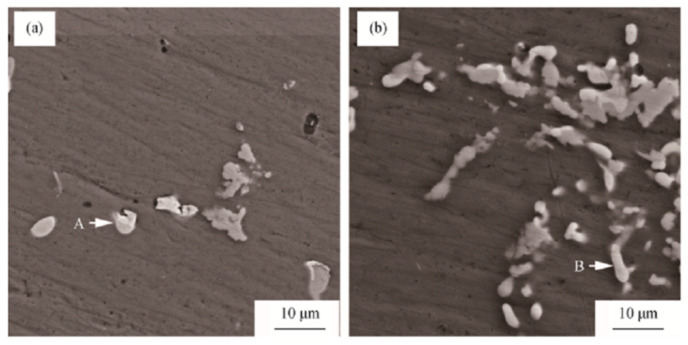
SEM images of homogenized ingots: (**a**) 7055; (**b**) 7055-0.25Sc. Adapted with permission from ref. [87]. 2019 Liu et al.

The morphologies of 7055 and 7055-0.25Sc ingots after homogenization are shown in Figure 19 [87]. The energy dispersive X-ray spectroscopy (EDS) spectrum of particle A, Figure 12a shows that the particle is in the θ phase. The EDS results measured by particle B in Figure 19b correspond to w (AlCuSc) phase. In the Al-Cu-Sc system with high copper content, Sc atoms diffuse to the θ phase, resulting in the transformation of θ phase into w phase during homogenization [88,89]. When the total mass fraction of Sc and Zr exceeds 0.45 wt%, w phase, primary Al_3_ (Sc, Zr) phase and refined grains are insufficient, and the mechanical properties of 7055-xZrySc rolled plate treated with T6 deteriorate, thus 7055-0.25Sc rolled plate shows the optimal mechanical properties in the prepared alloy.

### 4.3. Forming Process

The conventional deformation processes of 7xxx alloy, including equal channel angular pressing (ECAP), asynchronous rolling (ASR), high pressure torsion (HPT), accumulative roll-over (ARB) and reciprocating extrusion (CEC), include rolling, extrusion, and forging [79]. Additive manufacturing is a technology of manufacturing solid parts by the gradual build-up of materials. Powder metallurgy (PM), and severe plastic deformation (SPD) are well-known technological solutions used to achieve properties [90,91]. In recent years, some high energy beam welding, including electron beam welding (EBW) and laser welding (LBW), and friction stir welding (FSW) have been applied to weld 7xxx aluminum alloy. Friction stir welding (FSW) is a solid-phase joining technology, rapidly developed in recent decades. The biggest difference from other welding processes mentioned above is that FSW has a low welding temperature, and no welding pool is generated during welding [92].

Whether using a traditional forming process or a new additive manufacturing process, what is required is to research the elimination of manufacturing defects through the deformation process, and the production of ultra-fine grain microstructure resulting from work hardening and fine-grain strengthening to improve the mechanical properties of materials and increase the precipitation density of η′ phase, which is also a far-reaching method used for strengthening alloys in industry.

Asynchronous rolling can be called snake rolling according to the process characteristics. Asynchronous rolling introduces shear strain and increases the deformation of the core plate, which can effectively solve the serious uneven strain distribution of aluminum alloy plate in the symmetric rolling process. Xu et al. [93] found that the strength performance of the alloy plate increased with the increase in the speed ratio, and the elongation and fracture toughness decreased. When the speed ratio is the same, with the increase in offset, the strength of serpentine rolled sheet decreases, and the elongation and fracture toughness increases significantly. In the condition where offset is 10 mm and speed ratio is 1:1, the crack unit nucleation energy of the serpentine rolled sample increases by 14~36%. Xia [94] simulated the asynchronous rolling of magnesium alloy sheets. The results show that under the condition of the same total deformation, the multi-pass asynchronous rolling with small speed ratio cannot only effectively obtain large shear strain accumulation, but also the distribution of equivalent strain is more uniform than that of large speed ratio rolling, and the multi-pass alternating asynchronous rolling can significantly improve the high uniformity of thick plates.

**Figure 20:**
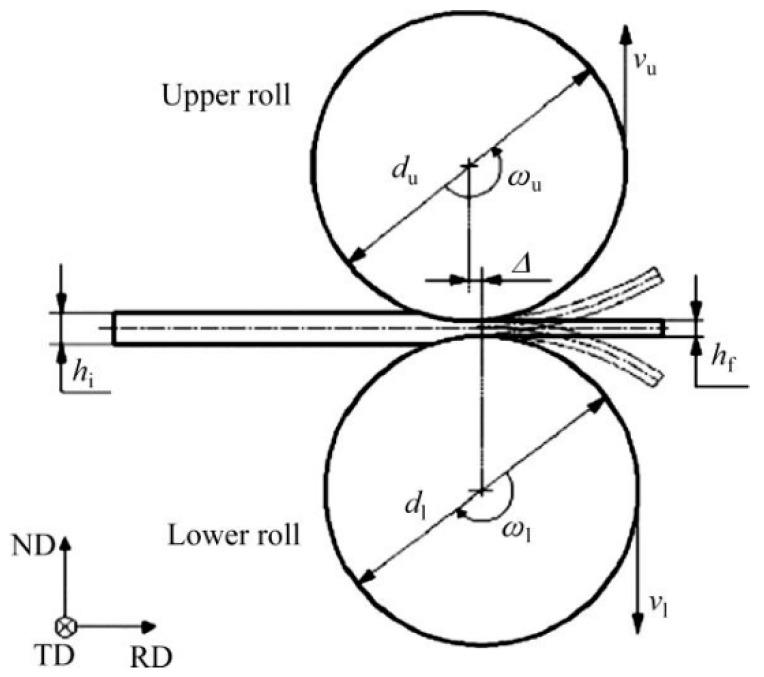
Schematic diagram of snake rolling. Adapted with permission from ref. [95]. 2016 Li et al.

Figure 20 shows the schematic diagram of snake rolling [95]. As shown in the figure, the lower roller moves a distance (Δ) horizontally relative to the upper roller, and the peripheral speed ratio (vu/vl) between the upper and lower rollers can be adjusted by altering the speed ratio (ωu/ωl) or diameter (du/dl). Extensive studies have shown that the snake rolling method can, not only refine the grain, but also change the crystal structure [96,97,98].

### 4.4. Heat Treatment Method

The purpose of heat treatment is to adjust the method suitable for the material properties according to grain size, solute atoms, GBPs and microstructure of precipitates, mainly including solid solution treatment and artificial aging treatment. Methods such as single-stage and multi-stage solution, peak aging, excessive aging, multi-stage aging and regression re-aging are widely applicable.

#### 4.4.1. Solution Treatment

**Figure 21:**
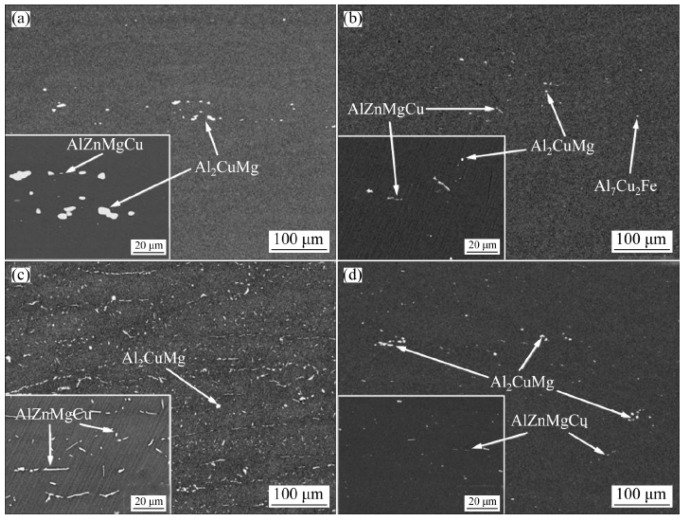
Second-phase distribution after different solid solution treatments in 7055: (**a**) SST; (**b**) EST; (**c**) HTPP; (**d**) MST. Adapted with permission from ref. [105]. 2021 Zhang et al.

Solid solution treatment, known as quenching, is the basis of strengthening heat treatment (quenching and aging) of aluminum alloy strengthened by heat treatment [99]. Solid solution treatment is classified into single-stage solid solution treatment (SST), enhanced solid solution treatment (EST), high temperature pre-precipitation (HTPP) and multi-stage solid solution treatment (MST) [100,101,102]. The objective of solid solution is to dissolve the alloying elements into the aluminum matrix to achieve a well strengthening effect in the subsequent aging precipitation stage. The single-stage temperature and constant-temperature multi-stage solid solution method can effectively alleviate the coarsening of alloy grains at high temperature. In multi-stage solution treatment (MST), the heating and holding process is divided into several stages from low temperature to high temperature, which can significantly improve the comprehensive performance of aluminum alloy. Feng et al. [100] used (470 °C,1H) + (480 °C,1H) two-stage solid solution treatment for 7A55 alloy to eliminate the non-equilibrium of the surface layer of 7A55 aluminum alloy thick plate η′. With high phase volume fraction, the heterogeneity of hardness and conductivity of 7A55 aluminum alloy thick plate can be improved. The commonly used solid solution process of 7xxx series aluminum alloy is [103,104] 450 °C/2 h + 460 °C/2 h + 470 °C/2 h + 480 °C/2 h. After water-cooled solid solution strengthening and 121 °C/12 h aging treatment, the alloy can obtain better mechanical properties, and the strength, hardness and plasticity become superior. Figure 21 [105] shows the second-phase distribution after different solid solution treatments. It can be seen that the solution treatment will significantly affect the grain size of the alloy and the solid solution degree of the solute atoms, thus changing the precipitation kinetics of the alloy in the subsequent aging process, and the number and density of the aging precipitates will ultimately determine the overall performance of the alloy.

#### 4.4.2. Aging Treatment

The traditional 7xxx series aluminum alloy has high strength after peak aging (T6) treatment, but its SCC performance is low. Although over-aging treatment can greatly improve the corrosion resistance of the alloy, the strength loss is large. In order to solve this problem, regression re-aging (RRA) is introduced to balance the properties of materials [106,107]. Zheng et al. [108] improved T6176 intermittent aging system for 7475 aluminum alloy thick plate, and obtained the strength equivalent of T6 state and the local corrosion performance better than that of T73. Aging is a non-isothermal process. Ding [109] identified the effects of T6, four-stage aging, three-stage aging and T74 on thermal stability by tensile test, transmission electron microscopy (TEM) and atomic probe tomography (APT). The results showed that the tensile strength of the four-stage aging sample decreased by only 5.05% after thermal exposure at 120 °C for 500 h. For the regression re-aging (RRA) treatment with stricter control of heat treatment time, the temperature of this process is high, and the precipitated phase will experience multiple reactions of dissolution, nucleation, growth and coarsening, respectively, or simultaneously in different regression temperature ranges. The hardness of 7xxx series aluminum alloy is higher than that of T651 (HV180) when the RRA heat treatment is carried out at a relatively low temperature (180 °C) and with a short degradation period. However, with the increase in temperature, the hardness decreases with the increase in the degradation period. After RRA treatment at 180 °C for 4 min, the hardness is the highest. The hardness of 7xxx series aluminum alloy is higher than that of T651 (HV180) when RRA heat treatment is carried out at a relatively low temperature (180 °C) and with a short degradation period, but with the increase in temperature, the hardness decreases with the increase in degradation period, and the hardness reaches the highest after RRA heat treatment at 180 °C for 4 min [79,110]. Liao [111] carried out different combinations of pre-aging, regression treatment and re-aging systems on 7055, and obtained the optimum RRA system for actual production in the factory, which is 120 °C/24 h + 175 °C/1.5 h + 120 °C/24 h, in which the heating rate is 35 °C/h, and the cooling method is air cooling. The results show that the pre-aging temperature has more effect on the size of the precipitated phase than the re-aging temperature. After RRA treatment [112], the material will be over-aged. During the re-aging process, a new precipitate occurs in the η′ phase, while the coarse η′ precipitate grows and transforms into the η phase. It shows the regression stage undergoes heating, heat preservation, cooling and other processes, and there are many kinds of precipitates in the crystal, and the evolution law is sophisticated. However, the precipitation is a single type of equilibrium. Therefore, for the RRA treatment, the grain boundary precipitates can be selected as the object, and the non-isothermal regression kinetic model can be established to guide the precise regulation of aging process parameters.

## 5. Challenges and Conclusions

The biggest challenge for aluminum alloy today is that in order to greatly improve the corrosion resistance of the alloy, the strength loss is large. Both cannot be improved at the same time. Furthermore, due to the low melting point of aluminum alloy, its high temperature properties need to be improved. More attention should be paid to how to simultaneously improve the corrosion resistance and strength at the same time. Additive manufacturing of aluminum alloy, which has the characteristics of uniform chemical composition, high density, open forming environment, unlimited size of forming parts and high forming rate, is also the focus of future research. The optimization of alloy composition design and improvement of heat treatment method are the specific means to improve the comprehensive performance of 7xxx series aluminum alloy. The break-through of aluminum alloy composition design-microstructure characterization theory and research method promotes the development process of high strength aluminum alloy. Based on the research content, the research directions and methods of 7xxx series aluminum alloys in the future are determined as follows:

- To investigate the total amount and proportion of the main alloy elements and the action law of microalloying elements, Thermo-Calc, VASP, Factsage, Materials Studio and other software can be employed to integrate theoretical calculation, simulation and experiment. The research and development, performance improvement and product development period of alloys can be considerably shortened, and the efficiency can be improved immensely.
- Under the guidance of the calculation results of the first-principles theory, the microstructure-property characterization method of aluminum alloy was continuously improved according to the relationship among alloy composition, process, microstructure and property. The new principle and characterization method of the process-microstructure-property correlation of materials was developed to explore the characteristic microstructure 7xxx series aluminum alloy materials with high static strength, high strength, heat resistance, high toughness, damage resistance, low density, low quenching sensitivity, and high comprehensive performance, at the cutting edge.
- The η′ Phase is the only strengthening phase in the alloy, in order to increase the volume fraction of η′ precipitates and modify strength, rare earth elements should be added to the alloy, while controlling the Mg/Zn mass ratio in the range of 5:2–7:1 can refine the precipitates and improve the strength of the alloy and keep the alloy corrosion resistant.
- Sc rare earth element is currently the most significant alloying element, Al_3_(Sc,Zr) particles with L1_2_ structure precipitated at any time into coherent Al_3_(Sc,Zr) particles with D0_23_ structure can effectively hinder recrystallization, inhibit grain growth, and improve the mechanical properties of the alloy. Er and Sc rare earth elements are relatively low, and the eutectic point composition of Sc is the lowest, w (Sc)% ≈ 0.3%, w (Er) ≈ 1.0%. Therefore, it can be perceived that the properties of 7xxx series aluminum alloy with Sc addition will be remarkably improved. Er is much cheaper than Sc; consequently, Er is a potential rare earth element to replace Sc.
- The width, GBPs sum, distribution and continuity of the average PFZ determine the overall performance of the material; the purpose of different homogenization, multi-stage aging and RRA heat treatment is to adjust the method suitably to achieve material properties, according to grain size, solute atoms, GBPs and microstructure of precipitates.
- Although the heat treatment process of 7xxx series aluminum alloy requires controlling time lapses in the process of heating, holding and cooling, and there are many species precipitates in the crystal and the evolution mechanism is heterogeneous, the precipitated strengthening phase is a single type of equilibrium η′ phase. Therefore, for the heat treatment process, the grain boundary precipitate can be selected as the object, and the non-isothermal regression kinetic model was established to guide the accurate regulation of aging process parameters. More attention should be paid to how to increase the volume fraction of η′ precipitates and modify the comprehensive performance of the material by the regression re-aging method.
- Asynchronous rolling introduces shear strain and increases the deformation of the core plate, which can effectively solve the serious uneven strain distribution of aluminum alloy plate in the symmetric rolling process. The snake rolling method can not only refine the grain, but also change the crystal structure, therefore, it can be popularized for use.

## Figures and Tables

**Table 1:** Summary of alloy elements on relevant properties of Al-Zn-Mg-Cu [3,6,8,9,10,11,12,13,14,15,16,17,18].

Element	Precipitate	Content (ω%)	Main Effect
Zn + Mg	η (MgZn_2_); T (Al_2_Mg_2_Zn_3_)	0.0–10 (Zn ≥ 0.9)	Increased tensile strength; heat treatment effect
		Zn + Mg ≥ 10%	Decreased conductivity, fracture toughness, stress-corrosion resistance, and spalling-corrosion resistance
Mg	β (Al_8_Mg_5_)	0.0–4.0 (Mg ≥ 0.0)	Reduced welding crack tendency
Mg + C	S (CuMgAl_2_)	Zn/Mg > 2.2 Cu > Mg	Improved alloy strength
Cu	θ (CuAl_2_)	≤3	Improved corrosion resistance
			Expanded the stable temperature range of GP zone, improved the tensile strength, plasticity and fatigue strength
Cr	Incoherent E (Al_18_Cr_2_Mg_3_)	≤0.35	Nucleation and precipitation of coarse equilibrium phase
	(CrMn)Al_12_; (CrFe)Al_7_	0.1–0.2	Fine grain strengthening; inhibits recrystallization nucleation and growth; Improve anti SCC ability
Mn	Al_6_Mn	0.2–0.4	Improved maximum tensile strength and fracture toughness, performance of low cycle fatigue, quenching sensitivity
	Al_20_Cu_2_Mn_3_	>0.4	Reduced the number of strengthening phases
Zr	Al_3_Zr	0.05–0.16	Improved the strength, toughness, aging effect and corrosion resistance of the alloy
Ag		0.16	Promoted the formation of GP region and transition phase; delayed the over-aging of the alloy
Co	(Co,Fe)Al_9_, Co_2_Al_9_	0.05≤ ≥0.2	Improved the hardenability; retention of subcrystalline structure
Ti		0.01–0.08	Refined grain, improved casting properties
Er	Al_3_Er; Al_8_Cu_4_Er	0.1–0.15	Improved the toughness; hardenability; dimples appear
Sc	Al_3_Sc	0.1–0.4	Grain refinement; recrystallization inhibition
Sc + Zr	Al_3_(Sc,Zr)	0.6	Improved anti SCC ability
Y	Al_3_Y	0.3	Improved the hardness, tensile strength, elongation
Gd	Al_3_(Gd,Zr)	0.11	Hindered dislocation; grain boundary movement
Si	Mg_2_Si; AlFeMnSi	≥0.15	Reduced plasticity and fracture toughness
Fe	Al_6_FeMn	≥0.15	Reduced plasticity and fracture toughness

**Table 2:** 7xxx series aluminum alloy grade and main chemical composition (w%) [20,21,23,24,25,26].

Allloys	Si	Fe	Cu	Mn	Mg	Cr	Zn	Ti	Zr	Other		Al
										Each	Total	
7A01	0.30	0.30	0.01	-	-	-	0.9–1.3	-	-	0.03	-	Bal.
7A03	0.20	0.20	1.8–2.4	0.10	1.2–1.6	0.05	6.0–6.7	0.02–0.08	-	0.05	0.10	Bal.
7A04	0.50	0.50	1.4–2.0	0.20–0.60	1.8–2.8	0.10–0.25	5.0–7.0	0.10	-	0.05	0.10	Bal.
7A05	0.25	0.25	0.20	0.15–0.40	1.1–1.7	0.05–0.15	4.4–5.0	0.02–0.06	0.10–0.25	0.05	0.15	Bal.
7A09	0.50	0.50	1.2–2.0	0.15	2.0–3.0	0.16–0.30	5.1–6.1	0.10	-	0.05	0.10	Bal.
7A10	0.30	0.30	0.50–1.0	0.20–0.35	3.0–4.0	0.10–0.30	3.2–4.2	0.10	-	0.05	0.10	Bal.
7A19	0.30	0.40	0.08–0.30	0.30–0.50	1.3–1.9	0.10–0.20	1.5–5.3	-	0.08–0.20	0.05	0.15	Bal.
7A33	0.25	0.30	0.25–0.55	0.05	2.2–2.7	0.10–0.20	4.6–5.4	0.05	-	0.05	0.10	Bal.
7A52	0.25	0.30	0.05–0.20	0.20–0.50	2.0–2.8	0.15–0.25	4.0–4.8	0.05–0.18	0.05–0.15	0.05	0.15	Bal.
7003	0.30	0.35	0.20	0.30	0.50–1.0	0.20	5.0–6.5	0.20	0.05–0.25	0.05	0.15	Bal.
7020	0.35	0.20	0.20	0.05–0.50	1.0–1.4	0.10–0.35	4.0–5.0	-	0.08–0.20	0.05	0.15	Bal.
7022	0.50	0.50	0.50–1.0	0.10–0.40	-	0.10–0.30	4.3–5.2	-	-	0.05	0.15	Bal.
7050	0.12	0.15	2.0–2.6	0.10	1.9–2.6	0.04	5.7–6.7	0.06	0.08–0.15	0.05	0.15	Bal.
7075	0.40	0.50	1.2–2.0	0.03	2.1–2.9	0.18–0.28	5.1–6.1	0.02	-	0.05	0.15	Bal.
7475	0.10	0.12	1.2–1.9	0.06	1.9–2.6	0.18–0.25	5.2–6.2	0.06	-	0.05	0.15	Bal.

**Table 3:** Properties of major rare earth elements and their properties in aluminum [20,24,36,37,38].

Element	HV	Atomic Radius (nm)	Relative Difference with Al Atomic Radius (%)	Electronegativity	Electronegativity Difference with Al	Melting Point of Pure Aluminum and Eutectic Temperature Difference (°C)	Eutectic POINT Composition (w(RE)%)
Er	700	0.1757	23	1.2	0.3	5	1
Sc	850	0.1641	14.8	1.3	0.2	5	0.3
Y	600	0.1803	26.2	1.2	0.3	10	3.3
La	400	0.1877	31.4	1.1	0.4	20	2.5
Ce	250	0.1824	27.6	1.05	0.45	20	2
Nd	350	0.1522	27.5	1.2	0.3	20	7.4
Sm	450	0.1802	26.1	1.2	0.3	28	1.5
Eu	-	0.2041	42.8	1.1	0.4	-	-
Gd	550	0.1801	26	1.2	0.3	17	2
Tb	600	0.1783	24.8	1.2	0.3	16	1.8
Dy	550	0.1775	24.2	1.2	0.3	24	8.2
Ho	600	0.1767	23.7	1.2	0.3	13	2.6
Tm	650	0.1747	22.3	1.2	0.3	16	1.7
Yb	250	0.1939	35.7	1.1	0.4	35	4

**Table 4:** Morphology and structure characteristics of main precipitates in 7xxx series aluminum alloy [50,51,52,53,54].

	Precipitation	Crystal Structure	Lattice Constant/nm			Morphology And Crystal Orientation	Precipitation Temperature/°C
			a	b	c		
Metastable phase	H′ (MgZn_2_)	Hexagonal system (L1_2_)	0.496	-	1.403	semi-Coherent, (0001)_η′_//{111}_Al_; (11–20)_η′_//<112>_Al_; (10–10)_η′_//(110)_Al_; habit plane(111)_Al_	120–250
	η_p_ (Al_2_Mg_2_Zn_8_)	Hexagonal system	0.496	-	0.935	semi-Coherent, (0001)_ηp_//{111}_Al_; (11–20)_ηp_//<112>_Al_; (10–10)_ηp_//(110)_Al_	300–400
	T′ (Al_2_Mg_2_Zn_3_)	Cubic system	500	-	-	Incoherent; Equiaxed Polytopic	471–476
	S′ (Al_2_CuMg)	Orthorhombic system	0.4012	0.9265	0.7124	Incoherent; Bar shape	456~482
Stable phase	η (MgZn_2_)	(D0_23_)	0.516–0.522	-	0.849–0.855	Incoherent; 13 orientation relationship; classic orientation relationship (0001)η//{111}_Al_, (10–10)η//{110}_Al_	150–476
	S (Al_2_CuMg)	Orthorhombic system	0.4	0.025	0.715	semi-Coherent	400–494
	T (Al_2_Mg_2_Zn_3_)	Cubic system	1.416	-	-	Coherent, (060)T//(111)_Al_;(103)//(2–20)_Al_;	Low temperature precipitation: 174
	θ (CuAl_2_)	Tetragonal system	0.607	-	0.487	Coherent	451; Low temperature precipitation: 80

## Data Availability

Not applicable.
